# The Phytochemical Characterization of a Cili (*Rosa roxburghii*) Fruit Low-Temperature Extract with Hepatoprotective Effects

**DOI:** 10.3390/foods14081301

**Published:** 2025-04-09

**Authors:** Rifeng He, Ziling Lian, Zhongjun Cheng, Yang Liu, Xiaoyan Peng, Yong Wang, Hang Ma, Xue Zhou, Fahuan Ge

**Affiliations:** 1 School of Pharmaceutical Sciences, Sun Yat-sen University, Guangzhou 510006, China; 2 Bijie Institute of Traditional Chinese Medicine, Bijie 551700, China; 3 Bioactive Botanical Research Laboratory, Department of Biomedical and Pharmaceutical Sciences, College of Pharmacy, The University of Rhode Island, Kingston, RI 02881, USA

**Keywords:** cili (*Rosa roxburghii* Tratt), homogenate assisted, high-pressure disruption, low-temperature extracts, liver fiber, liver injury

## Abstract

Cili (*Rosa roxburghii* Tratt) fruit is a nutrient-rich edible plant known for its antioxidant and hepatoprotective properties. However, conventional extraction methods often lead to the degradation of its bioactive compounds. In this study, we developed a low-temperature homogenate-assisted high-pressure disruption extraction (HHPD) method to obtain a phytochemically enriched cili fruit extract (HHPD-CFE). The chemical characterization of the HHPD-CFE showed that it contained higher levels of polyphenols, polysaccharides, and superoxide dismutase (SOD) than those in conventional squeeze extraction. The hepatoprotective effects of the HHPD-CFE were evaluated in oxidative stress-induced liver injury and hepatic fibrosis models. The HHPD-CFE mitigated oxidative damage by reducing malondialdehyde while enhancing SOD and glutathione activity. Additionally, the HHPD-CFE inhibited the activation of hepatic stellate cells (HSC-T6) and reduced collagen deposition, suggesting a protective role against liver fibrosis. These findings support that the HHPD-CFE is a promising botanical extract with enriched bioactive compounds and liver-protective properties. This study supports the potential application of optimized extraction techniques to preserve thermosensitive compounds and improve the efficacy of functional foods for liver health.

## 1. Introduction

Cili (*Rosa roxburghii* Tratt.; also known as chestnut rose or burr rose), a member of the Rosaceae family, is native to the eastern Himalayas, Tibet, and southwest China. Cili fruit has been traditionally consumed as edible fruit in the southwestern Chinese diet and used as folk medicine for centuries [1]. Historical records indicate its use as a traditional Chinese medicine dating back over 300 years. Cili fruit is a rich source of bioactive compounds including vitamin C, polyphenols, flavonoids, polysaccharides, and organic acids. Notably, the cili fruit is renowned for its natural abundance of superoxide dismutase (SOD), a potent enzymatic antioxidant. These active components contribute to the cili fruit’s overall biological effects including antioxidant, anti-inflammatory, antitumor, hypoglycemic, and hepatoprotective activities [2,3,4,5,6]. Despite its exceptional nutritional and medicinal value, fresh cili fruit’s perishable nature poses significant transportation and storage challenges. Conventional squeeze extraction (CSE) is a commonly used method for processing fresh cili fruit; however, this technique often leaves substantial amounts of bioactive compounds in the pomace, resulting in inefficient resource utilization. Other traditional extraction methods, such as hot-water extraction and ethanol reflux extraction, are also commonly used for natural product extraction. However, these methods are subject to several limitations for cili fruit extraction. For instance, the hot-water extraction, which is a high-energy-consumption and extended extraction process, may undermine the thermosensitive components of cili (such as vitamin C) [7]. An ethanol reflux extraction requires substantial solvent volume, which imposes environmental and safety concerns. It has been reported that the ethanol-based solvent extraction of cili may have a low yield and phenolic content [8]. Therefore, developing efficient and sustainable extraction technologies is imperative to enhance the recovery of cili’s bioactive constituents, thereby promoting both environmental sustainability and economic viability.

Homogenate-assisted high-pressure disruption (HHPD) extraction combined with freeze-drying is a recently developed extraction method [9]. This technique applies instantaneous high pressure and pressure drops to the liquid vehicle to create a strong impact at low temperatures, which results in the expansion and breaking of plant cells and increases the contact area between the active ingredient and solvent [10,11]. Compared with conventional extraction methodologies, the HHPD extraction technique may enhance extraction efficiency and reduce extraction time and energy consumption while maintaining low temperatures throughout the process, preventing the degradation of heat-sensitive active compounds. In a previous study, we used the HHPD method to extract phenolic acids in Japanese honeysuckles with a higher yield and shorter processing time than that in ultrasound-assisted extraction [9]. Furthermore, HHPD can be an effective extraction capability for compounds (e.g., astaxanthin) that are susceptible to thermal oxidation [12]. This demonstrates that HHPD can be a suitable approach for the extraction of thermosensitive components in cili fruit.

Chronic liver injury can lead to the excessive deposition of extracellular matrix, which contributes to several liver diseases including liver fibrosis, cirrhosis, and cancer. Thus, natural products with hepatoprotective effects that mitigate liver fibrosis are promising approaches to prevent the progression of severe liver diseases. Notably, cili fruit extracts have been shown to exert hepatoprotective effects in various liver conditions [13,14]. However, the anti-fibrotic potential of cili fruit extracts is unclear. Moreover, the hepatoprotective compounds in the cili fruit are not fully characterized. In the current study, we aimed to (1) optimize the HHPD extraction for a bioactive-enriched cili fruit extract (CFE); (2) analyze the phytochemical constituents of the CFE using ultrafast liquid chromatography with ion trap time-of-flight mass spectrometry (UFLC-IT-TOF/MS); and (3) evaluate the anti-fibrosis effects of the cili fruit extract in cell-based assays using liver fibrotic cells (HSC-T6).

## 2. Materials and Methods

Fresh cili fruits (variety: Gui Nong No. 5) from the same batch were collected from Bijie, Guizhou, China, in August 2023. The maturity of the cili fruits was assessed based on size and external color. After picking, the fruits were transported to the Sun Yat-sen University laboratory and stored at −20 °C. A voucher specimen is deposited at the School of Pharmaceutical Sciences, Sun Yat-sen University (CL-GN3-23). A high-pressure crushing extractor was purchased from Juneng Biology Technology Co., Ltd. (Guangzhou, China). L-ascorbic acid, citric acid, gallic acid, protocatechuic acid, proanthocyanidin B1, catechins, and rutin (purity ≥ 98%) were purchased from Shanghai Yuanye Biotechnology Co., Ltd. (Shanghai, China). Chromatographic-grade methanol and phosphoric acid were purchased from Merck (Darmstadt, Germany). Fetal bovine serum and Dulbecco’s modified Eagle medium (DMEM) were purchased from Thermo Fisher Scientific (Waltham, MA, USA). Cryptotanshinone was purchased from Aladdin Biochemical Technology Co., Ltd. (Shanghai, China). The CCK-8, malondialdehyde (MDA), superoxide dismutase (SOD), and glutathione (GSH) assay kits and N-acetylcysteine were purchased from Beyotime Biotechnology Co., Ltd. (Shanghai, China). The Masson staining kit was purchased from Solarbio Biotechnology Co., Ltd. (Beijing, China).

### 2.1. Extraction Process

#### 2.1.1. HHPD-Extraction of CFE

Cili fruits (20 g) were first washed and then wipe-dried, and the fruit seeds were removed. After crushing with a blender (FE2166, Fortune Star Group Limited, Hong Kong, China), the cili fruit was homogenized in distilled water (50 mL) at 10,000 rpm for 5 min. Then a high-pressure crushing extractor was used to procure the extraction solution. The extraction process was optimized with a solid–liquid (material–water) ratio of 1:20 at 4 °C for 20 min under a pressure of 100 MPa. Then, the extraction solution was freeze-dried to afford the cili fruit extract (HHPD-CFE). The extraction yield of the CFE was calculated with the following formula:

Yield (%) = M/m × 100

where M (g) is the weight of the HHPD extract, and m (g) is the weight of the cili fruit.

#### 2.1.2. CS-Extraction of CFE

The cili fruits (20 g) were washed, and the seeds were removed; they were then squeezed into juice using a screw extractor (JYZ-V919, Joyoung, Hangzhou, China) and centrifuged at 5000× *g* for 20 min at 4 °C (H1850R, Cence, Changsha, China). The supernatant was freeze-dried to obtain the conventional squeezed CFE (CS-CFE; 2.04 g).

### 2.2. Chemical Characterization of CFE

#### 2.2.1. Determination of Total Phenolics

The total polyphenol content of the CFE was determined with the Folin–Ciocalteu method with minor modifications [15]. Briefly, the 0.5 mL CFE extract solution was mixed with 2.5 mL of Folin–Ciocalteu reagent (10% *v*/*v*). Then, 2 mL 10% Na_2_CO_3_ solution and 5 mL water were added after incubation in the dark for 5 min and reacted for 1 h in the dark. The absorbance at 760 nm was determined. Gallic acid was used as standard (y = 4.4623x + 0.0313, R^2^ = 0.9995), and the concentrations were expressed as milligram gallic acid equivalent (GAE) per 100 g of extract dry weight (DW).

#### 2.2.2. Determination of Total Flavonoids

The total flavonoid content of the CFE was determined by the sodium nitrite–aluminum nitrate method with modifications [16]. The CFE extract solution (6 mL) was mixed with 1 mL of 5% sodium nitrite solution. Then, 1 mL 10% aluminum nitrate solution was added after incubation in the dark for 6 min and reacted for 6 min in the dark. Then, 10 mL 4% sodium hydroxide solution and 7 mL water were added before the absorbance at 510 nm was determined after 20 min. Rutin was used as an external standard (y = 0.9893x + 0.0159, R^2^ = 0.9992), and the concentrations were expressed as milligram rutin equivalent (RE) per 100 g of DW.

#### 2.2.3. Determination of Total Polysaccharides

The total polysaccharide content of the CFE was measured with the sulfuric acid–phenol method [17]. The CFE extract solution (0.4 mL) was mixed with 0.6 mL water. Then, 1.0 mL phenol solution and 5.0 mL concentrated sulfuric acid were added and reacted for 10 min in the dark. The absorbance was determined at 490 nm after 20 min at 30 °C. Glucose was used as the external standard (y = 12.923x + 0.0159, R^2^ = 0.9991), and the concentrations were expressed as milligram glucose equivalent (GE) per 100 g of DW.

#### 2.2.4. Measurement of SOD

The SOD content of the CFE was determined by measuring the enzyme activity with the nitroblue tetrazolium (NBT) method using a total SOD assay kit [18]. The assay reagents were added to the 96-well flat-bottom plates and incubated at 37 °C for 30 min, and then the absorbance at 560 nm was measured. The SOD inhibition rate (I) of the sample and the SOD activity were calculated with the following formula, and each sample was measured in three replicates.

I = ((*A*_1_ − *A*_2_) − (*A*_4_ − *A*_3_))/((*A*_1_ − *A*_2_)) × 100 (1)

SOD activity (U/mg of DW) = (I × *V*_1_ × *N*)/(50% × *V*_2_ × *W*) (2)

In Formula (1), *A*_1_ is the absorbance without sample; *A*_2_ is the absorbance without sample and reaction starting solution; *A*_3_ is the absorbance without reaction starting solution; *A*_4_ is the absorbance of the spiked sample. In Formula (2), *V*_1_ is the total volume of reaction solution (mL); *V*_2_ is the sample volume (mL); *N* is the sample dilution multiple; and *W* is the sample concentration (mg/mL).

#### 2.2.5. Detection of Characteristic Compounds in CFE by HPLC

A high-pressure liquid chromatography (HPLC) method was used for the simultaneous quantification of seven characteristic compounds including vitamin C, citric acid, gallic acid, protocatechuic acid, proanthocyanidin B1, catechin, and rutin in the CFE. Briefly, the chromatographic column was a Cosmosil C18 column (250 mm × 4.6 mm; 5 μm) with a flow rate of 0.8 mL/min, wavelengths of 210 nm and 368 nm, and a column at a temperature of 30 °C. The mobile phase A and B were 0.1% aqueous phosphoric acid and methanol, respectively. The gradient elution program was as follows: 0–10 min, 2–20% B; 10–28 min, 20–22%B; 28–30 min, 22–23%B; 30–35 min, 23–50%B; 35–45 min, 50%B; 45–55 min, 50–60%B.

### 2.3. Identification of Chemical Constituents by UFLC-IT-TOF/MS in HHPD-CFE

Ultra-fast liquid chromatography-ion trap time-of-flight mass spectrometry (UFLC-IT-TOF/MS) was used to study the chemical constituents of the CFE. The liquid chromatography conditions were optimized as follows: the column was a Cosmosil C18 column (250 m × 4.6 mm; 5 μm); the flow rate was 1 mL/min; the detection wavelength was the full wavelength; the column temperature was 30 °C; the mobile phase A was 0.1% formic acid, and phase B was methanol; and the gradient elution procedure is described in Section 2.2.5. The mass spectrometry conditions were set as follows: the ion source was electrospray ionization (ESI) with positive and negative ion detection modes; the mass spectrometry detection ranges were 100–1500 for MS *m*/*z*, 100–1200 for MS^2^ *m*/*z*, and 50–800 for MS3 *m*/*z*; the ion accumulation time was 10 ms; the IT vacuum was 1.6 × 10^−2^ Pa; the TOF vacuum was 1.3 × 10^−4^ Pa; the instrument temperature was 40.0 °C; the collision gas was argon; the nebulization gas was nitrogen with a flow rate of 1.5 L/min; the pressure of the drying gas was 96 kPa; the interface voltage of the ion source was 4.5 kV/−3.0 kV; the detector voltage was 1.60 kV; the temperature of the heating module was 200 °C; the temperature of the desolventization tube was 200 °C; the calibration method was automatic adjustment of the optimized voltage; and the calibration solution was CF_3_COONa. The mass number was calibrated using the external standard method. Post-column splitting was performed, and the split ratio was 1:3.

### 2.4. The Anti-Liver Injury and Anti-Liver Fibrosis Activities of HHPD-CFE

The BRL and HSC-T6 cells were cultured in DMEM high-glucose medium enriched with 10% fetal bovine serum and 1% double antibody, maintained at 37 °C and 5% CO_2_. Cells in the logarithmic growth phase were seeded into 96-well plates and treated with varying concentrations of the CFE. The cell viability was assessed using a CCK-8 kit. BRL cells were stimulated with H_2_O_2_ as a cell injury model. Following treatment, the effects of the CFE on the growth of injured BRL cells and the levels of several biomarkers including MDA, SOD, and GSH were measured. For HSC-T6 cells, a cell fibrosis model was established by stimulating the cells with transforming growth factor-beta 1 (TGF-β1; PeproTech, Waltham, MA, USA). After treatment with various concentrations of the CFE, their effect on the growth of fibrotic HSC-T6 cells was measured. Additionally, Masson staining and immunofluorescence staining were used to visualize the effect of the CFE on the collagen and collagen I deposition in HSC-T6 cells.

### 2.5. Data Analysis

Data are expressed as Mean ± SD of three replicates and statistically analyzed using IBM SPSS statistics 27.0 software (IBM Inc., Chicago, IL, USA); graphing was performed using GraphPad Prism 9.0.0 (GraphPad Software, Boston, MA, USA). The statistical significance was analyzed using an independent-sample t-test and one-way analysis of variance (ANOVA) (*p* < 0.05).

## 3. Results and Discussion

The experimental parameters of HHPD were optimized by single-factor and orthogonal tests. The optimal process conditions of HHPD were used to produce the CFE. At a low extraction temperature, the optimized HHPD had a high extraction rate (5.1 ± 0.4%), which was higher (*p* < 0.001) than that for the CSE method (Table 1 and Table 2), demonstrating the advantage of low-temperature and high-pressure crushing.

### 3.1. Phytochemical Characterizations of CFE

Cili fruits contain a large number of phenolic compounds with biological effects, but these are unstable during conventional extraction processes [19,20]. The total phenolic content of the HHPD-CFE was 23.6 g/100 g DW, which was significantly higher than that of the CS-CFE (19.4 g/100 g DW; *p* < 0.05). Compared with conventional thermal extraction, the HHPD technology maintains a low temperature during the whole extraction process, which can effectively avoid the decomposition of thermosensitive polyphenols. It has been reported that, compared with traditional heat treatment, high-pressure extraction can more effectively retain the structure of complex polyphenols [21]. In addition, HHPD uses hydrodynamic high pressure, which is different from continuously applying high pressure [9]. This technology can disrupt the plant cell walls during pressure rise and transient pressure release, thus releasing polyphenols from cells without undermining their stability [22].

Next, the analysis of the flavonoids, a subtype of polyphenols with promising biological activities [23], showed a higher total flavonoid content in the HHPD-CFE than in the CS-CFE (33.4 g/100 g DW vs. 23.0 g/100 g DW, respectively). The high-pressure extraction has been reported to achieve a higher yield of flavonoids than that in ultrasound-assisted extraction [24]. This is because the content of total flavonoids in cili fruit juice can be increased by pressured extractions, given that HHPD can disrupt plant cells, resulting in more total flavonoids being released.

Additionally, the polysaccharide component may contribute to the overall biological activities of plant extracts [25]. The cili fruit polysaccharides have attracted the attention of the food industry due to their functional properties and remarkable value. The contents of total polysaccharides in the HHPD-CFE and CS-CFE were 13.7 g/100 g DW and 10.2 g/100 g DW, respectively. Our results showed that high pressure had a mild effect on the extraction of polysaccharides, whilst the combination of low temperature and high pressure increased the content of soluble polysaccharides [26]. A similar observation was reported in a study on the extraction of polysaccharide content from large-leaf yellow tea by a high-pressure treatment [27].

### 3.2. SOD Activity in CFE

The antioxidant effects of cili fruit are partially attributed to its high amount of the antioxidant enzyme SOD. The HHPD-CFE showed a higher SOD activity (21,194.6 U/g DW) than the CS-CFE (12,245.4 U/g DW). It is possible that low temperature reduces the loss of SOD and that high-pressure treatment improves the surface hydrophobicity of SOD and reduces the α-helical fraction of SOD [28]. High-pressure processing may activate the activity of enzymes under specific environmental and conditions. It has been reported that the SOD activity of cili juice increased from 2217 to 2970 U/mL under a pressure of 100 MPa for 5–25 min, which is in agreement with results obtained from our current study [29].

### 3.3. Chemical Markers in CFE

In addition to the characterization of the overall phytochemical constituents including polyphenols, flavonoids, and polysaccharides, the specific chemical markers present in the HHPD-CFE were analyzed. An HPLC method was optimized to simultaneously determine the levels of vitamin C, citric acid, gallic acid, protocatechuic acid, proanthocyanidin B1, catechin, and rutin in the HHPD-CFE (Figure S1). This method was optimized by parameters including detection linearity, precision, repeatability, stability, and recovery (Table S1). The established method has desired linearity, precision, repeatability, and stability, which is suitable for the quantitative analysis of the seven representative compounds. The levels of the seven chemical markers in the HHPD-CFE are shown in Table 2. Vitamin C in the HHPD-CFE was 31.5 g/100 g DW, which was significantly higher than that of CS-CFE (24.0 g/100 g DW). The levels of citric acid, gallic acid, protocatechuic acid, proanthocyanidin B1, catechins, and rutin were 1.1 g/100 g DW, 1.5 g/100 g DW, 0.9 g/100 g DW, 0.9 g/100 g DW, 4.2 g/100 g DW, and 0.8 g/100 g DW, respectively. All of these markers were higher in the HHPD-CFE than in the CS-CFE. This is in agreement with the reported study showing that vitamin C is the most abundant antioxidant in the CFE. Catechins, proanthocyanidin B1, and gallic acid are also antioxidants in the CFE [30]. The high levels of these antioxidants supported that the HHPD method is favorable for the extraction of bioactive compounds in the CFE. It is noted that HHPD effectively reduced the degradation of vitamin C, which is unstable in aqueous solution. Similarly, the HHPD treatment increased the yield of chlorogenic acid in Lonicera japonica [9]. Thus, the HHPD-CFE had higher levels of flavonoids, polyphenols, polysaccharides, SOD, and vitamin C than the CS-CFE.

### 3.4. Characterization of Phenolics in HHPD-CFE by UFLC-IT-TOF/MS

The characterization of the HHPD-CFE was achieved by the UFLC-IT-TOF/MS method along with the comparison of published studies. While several compounds have been qualitatively and quantitatively analyzed via HPLC, it can only provide limited structural information about these compounds. Thus, we used LC-MS analyses to facilitate the further structural elucidation of compounds, particularly within the analysis of the complex matrix. The chromatograms and mass spectra were collected, and the total ion flow diagrams in positive and negative ion modes are shown in Figure S2. The peaks were qualitatively identified based on the accurate molecular weights, molecular formulas, fragment ion information, and cleavage patterns of the components and in combination with standards and the relevant literature. A total of 62 compounds (including isomers) were identified, and they included 30 phenolic components, 13 flavonoids, 6 triterpenoids, 5 organic acids, 6 amino acids, and 2 other chemicals in the HHPD-CFE. The retention time, molecular formula, fragmentation information, and compound names of all the components are summarized in Table 3.

### 3.5. The Cytoprotective Effects of HHPD-CFE in Liver Cells

**Figure 1:**
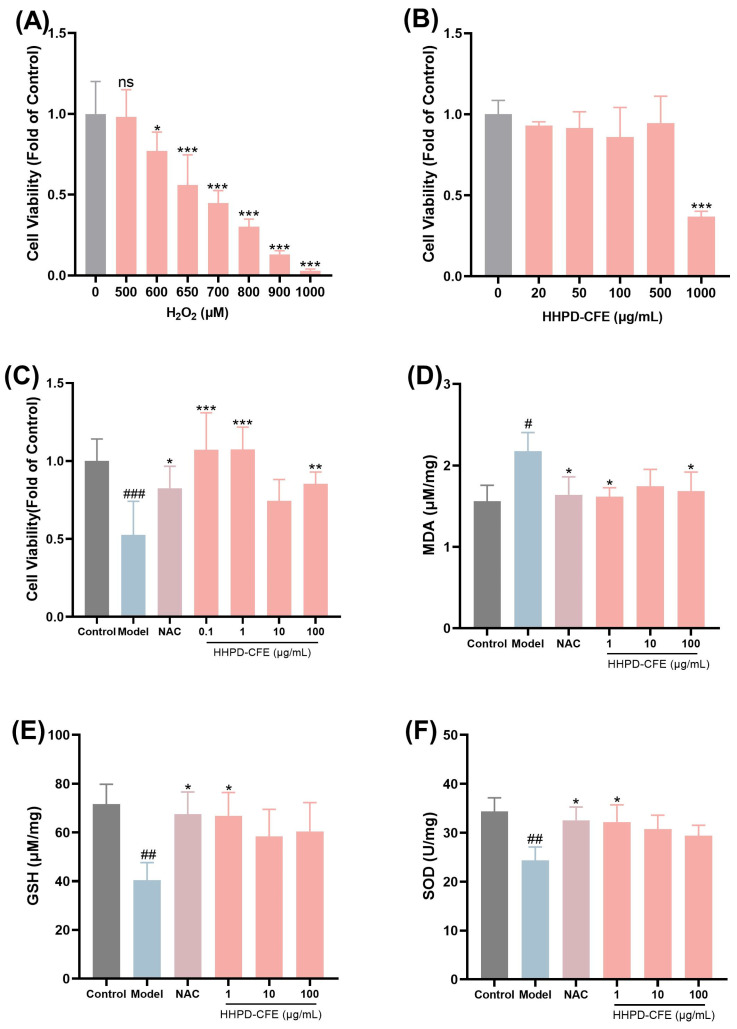
Effects of HHPD-CFE on H_2_O_2_-induced cellular injury in BRL cells. (**A**) Effect of H_2_O_2_ (500–1000 µM) on the viability of BRL cells. (**B**) Effects of HHPD-CFE on the viability of BRL cells. (**C**) Effect of HHPD-CFE on restoring the cell growth in BRL cells exposed to H_2_O_2_. (**D**–**F**) Effect of HHPD-CFE on MDA, GSH, and SOD in H_2_O_2_-treated BRL cells. Data are shown as mean ± SD (n = 3); # *p* < 0.05, ## *p* < 0.01, ### *p* < 0.001 (compared with the control group); * *p* < 0.05, ** *p* < 0.01, *** *p* < 0.001 (compared with the model group).

Given that the HHPD-CFE contains various antioxidants that may confer protective effects against liver conditions, we evaluated the cytoprotective activities of the HHPD-CFE in liver cells including BRL (rat fibroblast-like cells) and HSC-T6 (mouse immortalized hepatic stellate cells). First, a cellular liver injury model was constructed by inducing oxidative damage with hydrogen peroxide (H_2_O_2_) in BRL cells to evaluate the ameliorative effect of the HHPD-CFE. As shown in Figure 1, exposure to H_2_O_2_ (650 μM) for 24 h decreased the survival rate of BRL cells compared with the control group. N-acetylcysteine (NAC) was used as a positive control for this assay [31]. The damaged BRL cells were mitigated after the treatment with NAC (1 mM) and different concentrations of the HHPD-CFE (0.1, 1, and 100 µg/mL). Additionally, the intracellular MDA content was decreased (*p* < 0.05) by the HHPD-CFE treatment, whilst the SOD activity and GSH content were increased (*p* < 0.05), compared with that of the model group (Figure 1D–F). These data suggest that the HHPD-CFE exerted protective effects in hepatocytes against oxidative stress damage.

**Figure 2:**
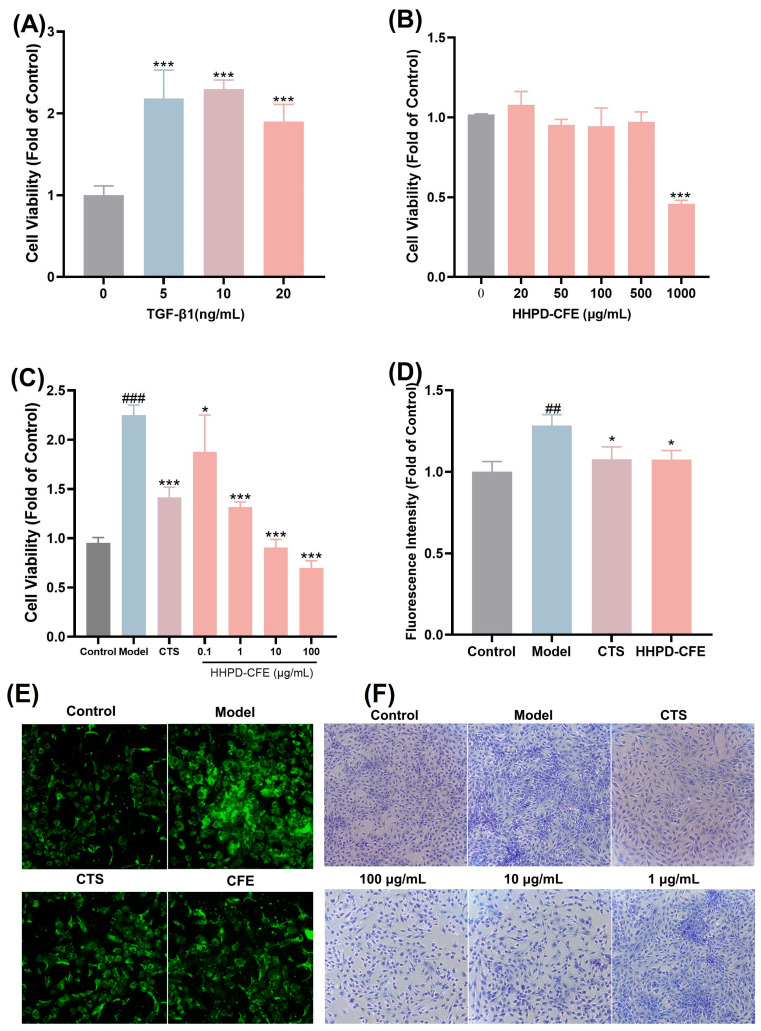
Effect of HHPD-CFE on TGF-β1-activated HSC-T6 cells. (**A**) A cellular fibrosis model with TGF-β 1-stimulated HSC-T6 cells was established. (**B**) Effect of HHPD-CFE on the viability of HSC-T6 cells. (**C**) Effect of HHPD-CFE on the proliferation of HSC-T6 cells activated by TGF-β1. (**D**,**E**) Effect of HHPD-CFE on TGF-β1-induced collagen I deposition in HSC-T6 cells. (**F**) Effect of HHPD-CFE on TGF-β1-induced fibrotic collagen deposition in HSC-T6. Data are shown as mean ± SD (n = 3); ## *p* < 0.01, ### *p* < 0.001 (compared with the control group); * *p* < 0.05, *** *p* < 0.001 (compared with the TGF-β1 group).

Liver fibrosis is a prolonged wound-healing response to chronic liver injury characterized by the accumulation of extracellular matrix (ECM). Hepatic stellate cells (HSCs) are the major ECM-producing cells in the injured liver. Thus, the inhibition of HSC proliferation can be a possible strategy for the intervention of liver fibrosis [32]. In the present study, the anti-liver fibrosis activity of the HHPD-CFE was evaluated by assessing its inhibitory effect on TGF-β1-activated mouse HSCs (HSC-T6 cells). As shown in Figure 2A,C, exposure to TGF-β1 induced the proliferation of HSC-T6 cells (*p* < 0.01). However, when exposed to different concentrations of the HHPD-CFE (0.1–100 µg/mL), the viability of activated HSC-T6 cells was reduced (*p* < 0.01) in a concentration- and time-dependent manner (Figure 2C). At a concentration of 1 μg/mL, the HHPD-CFE inhibited the proliferation of HSC-T6 cells (*p* < 0.001). Furthermore, the biomarkers of activated HSC-T6 cells including type I collagen and total collagen were increased (Figure 2D–F). Cryptotanshinone (CTS) was used as a positive control [33,34]. Compared with the model group, total collagen and collagen I synthesis were reduced (*p* < 0.01) in the cells treated with CTS (5μM). In contrast, the total collagen and collagen I synthesis in HSC-T6 cells were reduced by the HHPD-CFE at a concentration of 10 μg/mL. These data support that the HHPD-CFE can inhibit fibrosis in HSC-T6.

In traditional Chinese medicine, cili fruit is known for promoting food digestion. It is used for strengthening the spleen and ameliorating digestive issues including abdominal distension, diarrhea, and pain [35]. Although published studies support cili’s liver-protective effects, studies on the HHPD-CFE’s effects on liver stress and fibrosis are limited. Herein, we showed that the hepatoprotective effect of the HHPD-CFE might be attributed to its potent antioxidant activity (Table S2). In this study, we found that the HHPD-CFE inhibited H_2_O_2_-induced BRL cell injury and protected BRL cells by decreasing MDA levels and increasing the activities of SOD and GSH. In addition, the HHPD-CFE inhibited TGF-β-stimulated HSC-T6 cells and reduced collagen deposition. These data suggest that the HHPD-CFE can inhibit HSC-T6 cell activation to alleviate liver fibrosis. This is in agreement with reported studies showing that cili was effective against liver injury, and the mechanisms were associated with decreased oxidative stress, improved lipid metabolism through modulating nuclear receptor CAR-, PXR-, and Nrf2- pathways [13]. Additionally, cili fruit has been reported to prevent renal fibrosis and unilateral ureteral obstruction in rats by inhibiting the TGF-β 1/Smads signaling [36]. A combination of ginkgo and cili fruit extracts attenuated alcohol-induced liver injury, hepatic lipopolysaccharide binding signaling, and intestinal barrier dysfunction [37]. Together, data from our laboratory and others support the potential liver-protective effects of cili fruit. Moreover, our current study showed that cili fruit extract prepared using low-temperature and high pressure, i.e., HHPD-CFE, inhibits hepatocellular damage caused by oxidative stress and activation of hepatic stellate cells and may be a novel and effective therapeutic agent for liver injury and liver fibrosis.

## 4. Conclusions

In the current study, we developed a novel cili fruit extract (CFE) by using a homogenate-assisted high-pressure disruption extraction (HHPD) method. The HHPD method proved to be effective in preserving and enhancing the extraction of a wide range of bioactive components including polyphenols, flavonoids, polysaccharides, and superoxide dismutase (SOD), compared with conventional squeeze extraction. The chemical profiling of the HHPD-CFE was characterized by UFLC-IT-TOF/MS analysis, which revealed a diverse composition of phytochemicals. Additionally, our findings demonstrated that the HHPD-CFE exerted protective effects against oxidative stress-induced liver injury by reducing MDA levels and enhancing SOD and GSH activity. Furthermore, the HHPD-CFE inhibited hepatic stellate cell activation and collagen deposition, indicating its potential to prevent liver fibrosis. These results support that cili fruit extracts, particularly those obtained through low-temperature and high-pressure extraction techniques, have great potential to be developed as a functional food ingredient for liver protection. Overall, this study underscores the importance of optimizing extraction methodologies to maximize the stability of bioactive compounds in cili. The molecular mechanisms of the HHPD-CFE’s hepatoprotective effects are warranted to be further studied.

## Figures and Tables

**Table 1:** The contents of total polyphenols, total flavonoids, total polysaccharides, and SOD activity in HHPD-CFE and CS-CFE.

Ingredient	HHPD-CFE	CS-CFE	t	*p*
Yield (wt%)	10.2 ± 0.3	5.1 ± 0.4	23.3	<0.001
Total phenolics (g/100 g DW)	23.6 ± 1.5	19.4 ± 2.1	3.5	0.01
Total flavonoids (g/100 g DW)	33.4 ± 1.8	23.0 ± 1.7	8.3	<0.001
Total polysaccharides (g/100 g DW)	13.7 ± 0.2	10.2 ± 1.2	5.2	<0.01
SOD activity (U/g DW)	21,194.6 ± 571.4	12,245.4 ± 544.7	15.7	<0.001 ^a^

^a^ Represents a *t*-test that does not assume equal variance.

**Table 2:** Determination of the major chemical markers of HHPD-CFE and CS-CFE by HPLC analysis.

Ingredient	HHPD-CFE	CS-CFE	t	*p*
Vitamin C (g/100 g DW)	31.5 ± 0.6	24.0 ± 1.5	11.1	<0.001
Citric acid (g/100 g DW)	1.1 ± 0.1	0.9 ± 0.1	2.1	>0.05
Gallic acid (g/100 g DW)	1.5 ± 0.1	1.0 ± 0.1	5.9	<0.001
Protocatechuic acid (g/100 g DW)	0.9 ± 0.1	0.7 ± 0.1	3.5	0.01
Procyanidin B1 (g/100 g DW)	0.9 ± 0.3	0.5 ± 0.1	2.3	>0.05
Catechin (g/100 g DW)	4.2 ± 0.8	3.3 ± 0.2	2.1	>0.05 ^a^
Rutin (g/100 g DW)	0.8 ± 0.0	0.5 ± 0.1	4.9	<0.05 ^a^

^a^ Represents a *t*-test that does not assume equal variance.

**Table 3:** Tentative identification of phytochemicals in HHPD-CFE by UFLC-IT-TOF/MS.

No.	Rt (min)	Formula	*m*/*z*	Adduct Type	Ion Fragmentation	Error (ppm)	Chemical Name
1	2.55	C_6_H_14_N_4_O_2_	175.12	[M + H] ^+^	158.0216, 130.1014	−3.6	L-arginine
2	3.26	C_16_H_18_O_9_	353.09	[M − H]^−^	191.0514, 179.0623, 173.0923	−0.6	Chlorogenic acid
3	3.50	C_7_H_12_O_6_	191.05	[M − H]^−^	175.1374, 157.0557	−1.4	D-(-)-quinic acid
4	3.87	C_6_H_8_O_6_	179.04	[M + H] ^+^	141.0381, 129.0066, 110.9650	−2.6	Ascorbic acid
5	4.46	C_9_H_11_NO_3_	182.08	[M + H]^+^	147.0510, 136.0649	1.8	L-(-)-tyrosine
6	4.51	C_9_H_8_O_3_	163.02	[M − H]^−^	136.5035, 129.0305, 111.0161	1.5	p-Coumaric acid
7	4.82	C_13_H_16_O_10_	331.07	[M − H]^−^	271.0442, 169.0380, 125.0431	−0.2	Glucogallic acid
8	4.96	C_8_H_8_O_4_	169.05	[M + H]^+^	158.0243, 141.0328	2.8	Vanillin
9	5.16	C_6_H_8_O_7_	191.03	[M − H]^−^	189.0203, 173.0164	−2.7	Citric acid
10	5.97	C_13_H_16_O_10_	331.07	[M − H]^−^	271.0442, 169.0380, 125.0431	−0.2	Glucogallic acid
11	6.17	C_9_H_11_NO_2_	166.07	[M + H]^+^	120.074	−2.2	Phenylalanine
12	6.57	C_13_H_16_O_10_	331.07	[M − H]^−^	271.0442, 169.0380, 125.0431	−0.2	Glucogallic acid
13	6.79	C_7_H_6_O_5_	169.04	[M − H]^−^	140.9893, 124.7906	3.9	Gallic acid
14	6.93	C_13_H_16_O_10_	331.07	[M − H]^−^	271.0442, 169.0380, 125.0431	−0.2	Glucogallic acid
15	11.66	C_6_H_14_N_2_O_2_	147.20	[M + H]^+^	130.0671, 112.1516	1.1	L-lysine
16	11.82	C_11_H_12_N_2_O_2_	205.09	[M + H]^+^	188.0703, 170.0418, 146.0779	−3.1	Tryptophan
17	12.32	C_20_H_20_O_14_	483.08	[M − H]^−^	331.1121, 313.0624, 271.0343	0.6	b-D-glucopyranose,1,6-bis(3,4,5-trihydroxybenzoate) or isomer
18	15.22	C_9_H_10_O_5_	197.05	[M − H]^−^	151.0430, 125.0248	2.6	Syringic acid
19	16.51	C_7_H_6_O_4_	155.04	[M + H]^+^	137.0235, 109.0324	1.3	Protocatechuic acid
20	18.60	C_15_H_14_O_7_	305.06	[M − H]^−^	247.0570, 219.0522, 178.8766, 179.0447, 163.9822	0.6	(−)-Gallocatechin
21	19.23	C_15_H_14_O_7_	305.07	[M − H]^−^	289.0715, 279.1516, 267.0855,158.0302	−0.1	(−)-Epigallocatechin (EGC)
22	19.55	C_20_H_20_O_14_	483.08	[M − H]^−^	481.0583, 300.9917	0.6	b-D-Glucopyranose,1,6-bis(3,4,5-trihydroxybenzoate) or isomer
23	23.39	C_19_H_21_NO_6_	360.14	[M + H]^+^	331.1121, 313.0624, 271.0343	−1.5	(5R)-5-[(1S)-1,2-Dihydroxyethyl]-3,4-dihydroxy-2(5H)-furanone-1,1-diphenylmethanamine (1:1) (non-preferred name)
24	24.51	C_15_H_20_O_4_	265.15	[M + H]^+^	325.0783, 279.1636, 214.0829, 208.1057, 181.0699, 158.0385	1.8	Abscisic acid
25	27.87	C_27_H_22_O_18_	633.07	[M − H]^−^	247.1323, 217.1023, 161.1087, 158.0197	−0.3	Sanguiin H4
26	28.17	C_27_H_24_O_18_	635.09	[M − H]^−^	481.0604, 300.9969, 275.0149	0.5	1,3,6-Tri-O-galloylglucose
27	30.35	C_34_H_24_O_22_	783.07	[M − H]^−^	483.0244, 295.1465, 211.0867, 193.9747, 169.8194	1.6	Strictinin
28	32.30	C_21_H_20_O_1_	465.10	[M + H]^+^	481.0487, 300.9965	0.2	Quercetin-7-O-beta-D-glucopyranoside
29	33.69	C_12_H_18_O_8_	291.11	[M + H]^+^	301.0463, 151.0014	−2.1	Methyl 2,3,5-tri-O-acetyl-D-ribofuranoside
30	34.27	C_30_H_26_O_12_	577.13	[M − H]^−^	279.1302, 214.0895, 151.0341, 123.0414	2.2	Procyanidin B1
31	34.33	C_15_H_14_O_6_	289.07	[M − H]^−^	427.1061, 409.0956, 291.0857, 471.1479, 425.0881, 289.0685	−0.1	(−)-Catechin
32	34.62	C_30_H_26_O_12_	577.13	[M − H]^−^	245.0912, 203.0895, 179.0449, 161.0905	0.9	ProcyanidinB2
33	35.55	C_30_H_26_O_12_	577.13	[M − H]^−^	453.1606, 427.0961, 409.0792, 301.0616, 291.0862, 289.0693	3.2	Procyanidin B3
34	41.52	C_45_H_38_O_18_	865.20	[M − H]^−^	427.1025, 409.0848, 301.0670, 291.0837, 275.0394, 425.0845, 407.0809	0.8	Procyanidin C2
35	41.55	C_21_H_20_O_11_	435.09	[M + H]^+^	695.1335, 577.1316, 543.0864, 451.0935, 407.0689, 300.9994, 287.0525	−0.6	Quercitrin-3-O-D-xyloside
36	42.34	C_13_H_8_O_8_	291.02	[M − H]^−^	279.1542, 158.0262	−0.4	Brevifolincarboxylic acid isomer
37	43.07	C_27_H_22_O_18_	633.07	[M − H]^−^	247.0267, 219.0379, 203.0494, 191.1199	0.7	Sanguiin H4 or isomer
38	44.15	C_13_H_8_O_8_	291.02	[M − H]^−^	481.0604, 300.9969, 275.0149	−0.4	Brevifolincarboxylic acid
39	45.85	C_30_H_26_O_11_	561.14	[M − H]^−^	247.0872, 175.0202, 159.1008, 147.5432	0.8	Fisetinidol-(4α,8)-catechin
40	46.22	C_15_H_12_O_5_	273.07	[M + H]^+^	409.1684, 391.1559, 289.0820, 245.0814, 203.1727	−0.9	Dihydroapigenin
41	47.05	C_20_H_16_O_12_	447.06	[M − H]^−^	151.0488, 123.0605	0.7	Quercetin 3′-O-alpha-L-rhamnopyranoside
42	47.17	C_20_H_18_O_9_	401.10	[M − H]^−^	300.9967, 179.2039	−1.0	(Epi)catechin derivative
43	47.39	C_30_H_26_O_11_	561.14	[M − H]^−^	401.1233, 279.1556, 289.0508,	−0.4	Fisetinidol-(4α,8)-catechin
44	48.03	C_21_H_20_O_12_	465.11	[M + H]^+^	409.0689, 391.0705, 289.0602, 269.0557, 245.0814, 203.0631	3.3	Hyperoside
45	51.40	C_27_H_28_O_16_	609.14	[M + H]^+^	271.0505, 301.0723	−0.4	Quercetin 3-O[(X-O-3-hydroxy-3-methylglutaryl)-β-glucoside
46	54.56	C_16_H_12_O_7_	317.08	[M + H]^+^	301.0155, 179.2019	−3.8	beta-Rhamnocitrin or isomer
47	53.86	C_27_H_28_O_15_	593.14	[M + H]^+^	273.0558	0.6	Kaempferol 3-O-[(X-O-3-hydroxy-3-methylglutaryl)-β-galactoside]
48	62.20	C_27_H_28_O_6_	447.25	[M − H]^−^	257.0426, 241.0513	1.8	(6S,8S,8aS)-2-phenyl-6,7-bis(phenylmethoxy)-4,4a,6,7,8,8a-Hexahydropyrano[3,2-d][1,3]dioxin-8-ol
49	67.30	C_21_H_18_O_13_	479.08	[M + H]^+^	391.2793, 279.1617, 287.1353, 214.0882	−2.6	Quercetin 3-O-b-D-glucuronide
50	68.27	C_37_H_30_O_16_	729.15	[M − H]^−^	303.0207, 158.0354	1.3	Procyanidin B1 3-O-gallate
51	80.71	C_14_H_6_O_8_	301.00	[M − H]^−^	577.1340, 559.1054, 451.0978, 407.0765, 289.0725, 269.0910	−0.4	Ellagic acid
52	81.22	C_21_H_20_O_11_	447.10	[M − H]^−^	214.6353, 178.7923, 129.0305	−1.1	Quercitrin
53	81.30	C_27_H_30_O_16_	611.16	[M + H]^+^	432.1673, 405.3420, 151.0657	3.9	Rutin
54	81.37	C_34_H_24_O_22_	783.07	[M − H]^−^	303.0460, 301.0333	0.9	Cornusiin C
55	83.86	C_27_H_30_O_15_	593.13	[M − H]^−^	633.0728, 450.9200, 300.9983	1.7	Quercetin 3,7-di-O-rhamnopyranoside
56	92.30	C_27_H_28_O_16_	609.15	[M + H]^+^	489.1025, 301.0355, 271.0189	0.1	Kaempferol 3-O-[(X-O-3-hydroxy-3-methylglutaryl)-β-galactoside]
57	104.23	C_30_H_46_O_3_	455.35	[M + H]^+^	463.0562, 301.0301	0.5	Ursonic acid
58	105.28	C_30_H_48_O_4_	473.36	[M + H]^+^	437.2209, 409.3996, 391.3392, 231.1277, 201.1162, 191.1023	−0.2	Pomolic acid
59	105.35	C_30_H_48_O_5_	487.34	[M − H]^−^	455.2956, 409.3509, 369.1602, 318.1010, 201.1647, 191.1897	0.7	Tormentic acid
60	105.37	C_30_H_48_O_5_	487.34	[M − H]^−^		−2.2	Euscaphic acid
61	114.47	C_30_H_48_O_6_	503.34	[M − H]^−^	427.3343, 272.9934	0.9	Arjungenin
62	115.30	C_30_H_48_O_5_	487.34	[M − H]^−^	503.3486	−0.8	Asiatic acid

## Data Availability

The original contributions presented in this study are included in the article/Supplementary Material. Further inquiries can be directed to the corresponding authors.

## Supplementary Materials

The following supporting information can be downloaded at https://www.mdpi.com/article/10.3390/foods14081301/s1: Figure S1. The original HPLC chromatograms of the CFE and reference standards; Figure S2. Total ion flow diagram of HHPD-CFE in positive (A) and negative (B) ion mode; Table S1. The regression equation, linear range, R2, Rt, precision, repeatability, stability, and recovery of the HPLC; Table S2. Antioxidant activity of HHPD-CFE.

## Abbreviations

The following abbreviations are used in this manuscript:

HHPD	Homogenate-assisted high-pressure disruption extraction
SOD	Superoxide dismutase
HSC-T6	Hepatic stellate cells
CSE	Conventional squeeze extraction
CFE	Cili fruit extract
UFLC-IT-TOF/MS	Ultrafast liquid chromatography with ion trap time-of-flight mass spectrometry
MDA	Malondialdehyde
GSH	Glutathione
