# Supplementary material for: The Phytochemical Characterization of a Cili (Rosa roxburghii) Fruit Low-Temperature Extract with Hepatoprotective Effects
Source: Foods. 2025 Apr 9;14(8):1301. doi: 10.3390/foods14081301 (PMC12026329; doi:10.3390/foods14081301)
Supplement: Supplementary file 1 [file foods-14-01301-s001.zip › foods-3551261-supplementary.pdf]

Supplementary Materials

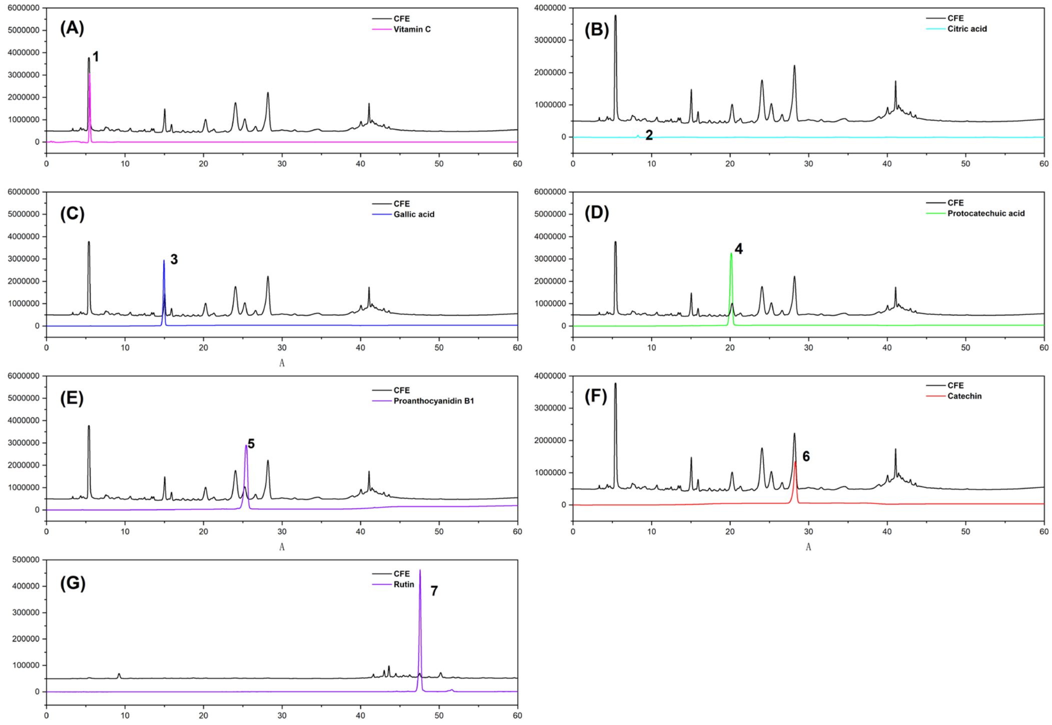

Figure S1. The original HPLC chromatograms of the CFE and reference standards(A-F:210nm; G:360nm)： (A)Vitamin C; (B) Citric acid; (C)Gallic acid; (D) Protocatechuic acid; (E) Proanthocyanidin B1; (F) Catechin; (G)Rutin

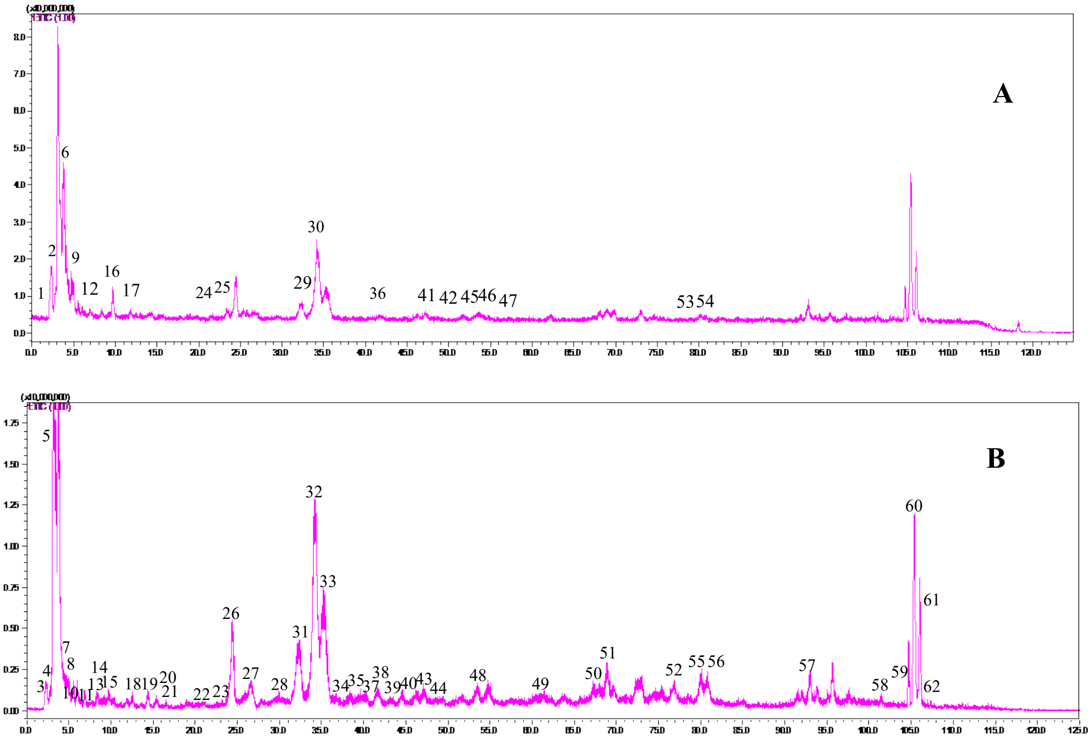

Figure S2. Total ion flow diagram of HHPD-CFE in positive (A) and negative (B) ion mode

Table S1. The regression equation, linear range, R2, Rt, Precision, Repeatability, Stability, and recovery of the HPLC.

Analyte	Regression equation	Linear range(mg/mL)	R^2	Rt[b] (min)	Precision (%)	Repeatability (%)	Stability (%)	Standard addition recovery (%)
Vitamin C	Y=2043472.8x+92882.8	0.102-1.02	0.9995	5.39	0.78	0.93	1.89	100.71
Citric acid	Y=8266688.4x-2443.3	0.1-1	0.999	7.86	0.96	1.11	0.91	101.23
Gallic acid	Y=30180006.7x-170442.5	0.01-0.08	0.9991	15.93	1.26	1.03	1.31	100.53
Protocatechuic acid	Y=68459630x-126526.3	0.01-0.1	0.9998	20.26	1.06	1.50	1.13	100.59
Proanthocyanidin B1	Y=77263841.1x-88806.1	0.01-0.1	0.9992	25.29	0.65	1.39	1.06	100.17
Catechins	Y=80146523.2x-1692827.2	0.025-0.4	0.9992	28.22	0.85	0.91	0.53	100.88
Rutin	Y=15162560.6x+27742.9	0.01-0.1	0.9997	45.49	1.08	1.86	0.64	99.53

b Rt refers to the retention time for each analyte.

Table S2. Antioxidant activity of HHPD-CFE.

Antioxidant experiments	HHPD-CFE	Vitamin C
DPPH[a]	23.24	10.85
ABTS[b]	0.154	0.118
OH[c]	0.265	0.105
FRAP[d]	3.895	13.417

[a]DPPH radical scavenging activity is expressed as IC50 of DPPH scavenging rate, and the unit is μ g/ml;

[b]ABTS radical scavenging activity is expressed as the IC50 of ABTS scavenging rate, and the unit is mg/ml;

[c]The OH radical scavenging activity is expressed as the IC50 of Oh scavenging rate, and the unit is mg/ml;

[d]FRAP value is expressed as the concentration of FeSO4 solution in the standard curve, and the unit is mmol/g.
